# Is Happiness a Possible Outcome for People With Schizophrenia?

**DOI:** 10.31083/AP46244

**Published:** 2026-02-02

**Authors:** Ilaria Riboldi, Giuseppe Carrà

**Affiliations:** ^1^School of Medicine and Surgery, University of Milano-Bicocca, 20900 Monza, Italy

## 1. Beyond Symptom Control: A Paradigm Shift in Schizophrenia Recovery

Schizophrenia has traditionally been associated with intense suffering, functional impairment, and social marginalization, and this perspective seems consistently accepted both within psychiatric care services and from a public health perspective [1]. Indeed, conventional approaches to outcome evaluation have predominantly focused on clinical factors, namely, symptom reduction, relapse prevention, and restoration of functional capacity [1]. Although these targets remain critical, such a framework reflects a self-circumscribed conceptualization of health that fails to adequately account for the subjective, lived experience of individuals [2]. However, a paradigm shift has recently emerged, focusing on subjective well-being across the recovery process [3]. This is consistent with the World Health Organization’s definition of health as “a state of complete physical, mental and social well-being and not merely the absence of disease or infirmity” [4]. Within this broader approach, well-being is not construed as a secondary by-product of symptom control, but can be regarded as a fundamental outcome of mental health care [3]. Alongside symptom remission and psychosocial functioning, mental well-being is a core goal of recovery that explicitly values the individual’s own understanding of what it means to live well [5, 6]. Indeed, people with schizophrenia also have the right to seek a life rich in meaning, satisfaction, and purpose [7].

## 2. Two Dimensions of Happiness 

Happiness has long been regarded as one of the most widely pursued human goals. Nevertheless, no single, universally accepted definition exists, and it is generally defined among researchers to be within the broader framework of mental well-being [8]. However, mental well-being is a multifaceted concept, typically comprising two interrelated, yet distinct, domains: hedonic well-being, which encompasses positive affect, life satisfaction, and happiness [9]; and eudaemonic well-being, which pertains to more deeply rooted aspects of psychological functioning, such as autonomy, environmental mastery, self-acceptance, purpose in life, and the presence of meaningful relationships [10, 11].

Research in this area has drawn upon multiple, though interrelated, frameworks to evaluate well-being. As a matter of fact, conceptual boundaries may be seen as somehow overlapping with those of “quality of life” (QoL), as both address the fundamental question of what it means to live well [12]. A similar overlap can be observed in schizophrenia, as well-being is defined and measured along a continuum from subjective self-reports to objective markers of material circumstances and functioning [13]. A merely environmental perspective, however, is insufficient to capture the components of happiness for individuals with schizophrenia. This challenge is further compounded by the inherently subjective nature of several clinical manifestations of the disorder, which frequently intersect with key dimensions of well-being [12].

## 3. Clinical Symptoms and Their Interplay With Well-being

**Fig. 1.**
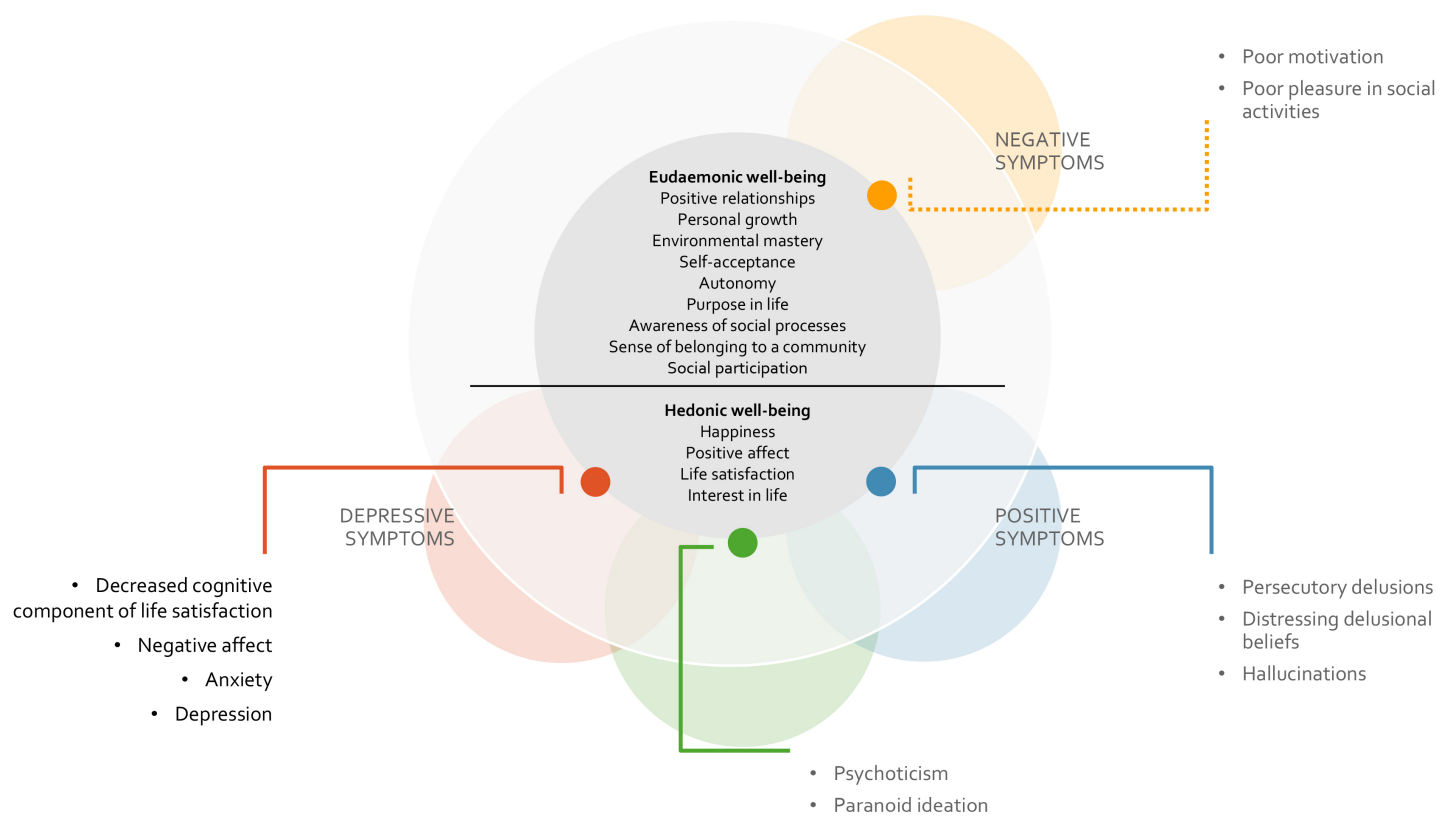
**Graphical representation of the hypothesized influence of specific features of positive, negative, and depressive symptoms on the two dimensions of well-being in individuals with schizophrenia**. The dashed line denotes the heterogeneity of findings regarding the impact of negative symptoms on eudaemonic features, with longitudinal study showing no significant association [24].

Schizophrenia’s core psychopathological features are conventionally dichotomized into positive and negative symptom domains, each demonstrating a measurable, albeit heterogeneous, association with diminished QoL [14, 15]. Positive symptoms, particularly delusional ideation and hallucinations, may profoundly compromise hedonic well-being through the induction of fear, psychological distress, and detachment from rewarding experiences [16, 17]. Moreover, dimensions such as psychoticism and paranoid ideation have been implicated in the attenuation of subjective well-being, although evidence has indicated that their detrimental effects are typically mediated by co-occurring depressive conditions [7, 18]. Indeed, depressive symptoms, frequently comorbid yet temporally and phenomenologically distinct from core psychotic processes [19], might further exacerbate hedonic impairments through a number of dimensions, including the cognitive component of life satisfaction, positive affect, and anxiety, along with depression [7, 20]. Conversely, negative symptoms seem to exert their principal effect on eudaemonic well-being, attenuating the ability to formulate and pursue goals, sustain volitional drive, and engage in socially and personally meaningful activities [21, 22, 23] (Fig. 1, Ref. [24]). Beyond the direct impact of symptom dimensions, recent research has suggested that pharmacological treatment, particularly when involving higher antipsychotic doses and associated side effects, may further compromise perceived happiness [25].

Although individuals with schizophrenia generally report lower subjective well-being than do healthy controls, within-group variability remains substantial [14, 26]. Indeed, symptoms can differ across illness stages and trajectories, as well as levels of cognitive impairment. In addition, trauma exposure and consequent dissociative features may further shape lived experience for a subgroup of patients, whereas comorbidities, including substance use disorders, anxiety, or depression, can differentially burden well-being [27, 28]. The predominance of cross-sectional designs used in research that addresses this issue has limited causal inference and has masked the dynamic interplay between symptoms and well-being [14, 26], underscoring the need for longitudinal studies to clarify how specific symptom dimensions shape distinct well-being domains, and to guide more targeted interventions. Furthermore, in the absence of a universal definition of subjective well-being, assessment tools should incorporate multiple indicators to identify those most at risk [14]. One widely used example is the Quality of Life Interview (QoLI), which has been validated in both clinical and research settings [29]. Notably, the European Schizophrenia Cohort (EuroSC) incorporated the QoLI into its design to assess well-being across large samples from France, Germany, and the United Kingdom [18, 30]. Authors identified items with face validity as indicators either of hedonic or eudaemonic well-being [31] and explored their associations with symptomatology over time [24]. Longitudinal analysis of the EuroSC data yielded compelling results: higher levels of positive symptoms were predictive of decreased hedonic well-being six months later, even after adjusting for depressive symptoms. In contrast, negative symptoms were not significantly associated with changes in either hedonic or eudaemonic well-being over time. Those findings suggested that although managing positive symptoms may be critical for alleviating emotional, hedonic distress, promoting deeper eudaemonic engagement likely requires broader psychosocial interventions [24].

## 4. Rethinking Outcome Targets: From Remission to Flourishing

Several critical considerations have merged from this evolving understanding. First, clinical practice must move beyond narrow symptom-based criteria for defining recovery. Instead, the ability to engage in fulfilling relationships, chase personal aspirations, and experience a sense of purpose should be moved to the foreground in outcome evaluations. Second, well-being must not be regarded as an optional aim of treatment, but as a core element of therapeutic planning. Interventions aimed at enhancing self-efficacy, social inclusion, and personal agency must be seen as essential components of mental health care. For instance, as positive symptoms have been shown to exert longitudinal effects on hedonic dimensions of well-being in schizophrenia, cognitive-behavioural strategies targeting these symptoms might also yield indirect benefits for hedonic features [32]. Similarly, resilience-building interventions aimed at reducing the enduring psychosocial burden of traumatic experience may facilitate recovery in individuals with trauma exposure, and targeted interventions for substance use and other comorbidities can represent crucial steps toward restoring well-being, particularly in vulnerable subgroups of people with schizophrenia. Moreover, continued refinement of assessment tools is imperative. Instruments capable of differentiating between hedonic and eudaemonic domains might enable clinicians and researchers to identify the particular areas of life most affected by specific symptoms, and to tailor interventions accordingly. Finally, a more robust empirical foundation is required to advance this field. Longitudinal studies, cross-cultural validations, and randomized controlled trials focusing explicitly on well-being outcomes, are urgently needed. Such research will be instrumental in informing future service design and in establishing well-being as a primary, not ancillary, outcome of schizophrenia care.

Overall, current perspectives emphasise that recovery from schizophrenia encompasses more than the remission of symptoms. A genuinely comprehensive model of care for people with schizophrenia must incorporate both hedonic and eudaemonic dimensions of well-being as legitimate and measurable treatment goals. As we move beyond the boundaries of symptomatology, our challenge is to construct systems of care that enable individuals not merely to survive, but to flourish.
